# Genome-Wide DNA Methylation Analysis of Hypothalamus During the Onset of Puberty in Gilts

**DOI:** 10.3389/fgene.2019.00228

**Published:** 2019-03-19

**Authors:** Xiaolong Yuan, Xiaofeng Zhou, Zitao Chen, Yingting He, Yaru Kong, Shaopan Ye, Ning Gao, Zhe Zhang, Hao Zhang, Jiaqi Li

**Affiliations:** ^1^Guangdong Provincial Key Lab of Agro-Animal Genomics and Molecular Breeding, National Engineering Research Center for Breeding Swine Industry, College of Animal Science, South China Agricultural University, Guangzhou, China; ^2^State Key Laboratory of Biocontrol, School of Life Sciences, Guangzhou Higher Education Mega Center, Sun Yat-sen University, Guangzhou, China

**Keywords:** genome-wide DNA methylation, hypothalamus, QTL of age at puberty in pigs, onset of puberty, gilts

## Abstract

Although selection of the early age at puberty in gilts will make for a favorable effect on the reproductivity of sow, a large proportion of phenotypic variation in age at puberty of gilts cannot be explained by genetics. Previous studies have implicated hypothalamic DNA methylation in the onset of puberty in mammals. However, the underlying molecular mechanism regarding the regulation of the onset of puberty has remained largely unexplored in gilts. Herein, the genome-scale DNA methylation of hypothalamus was acquired, using the reduced representation bisulfite sequencing, to compare and describe the changes of DNA methylation across Pre-, In- and Post-pubertal gilts. In this study, the average methylation levels of CpGs and CpHs (where H = C, T, or A) in CpG islands- and gene-related regions were gradually decreased in hypothalamic methylomes during the pubertal transition. Comparisons of Pre- vs. In-, In- vs. Post-, and Pre- vs. Post-pubertal stage revealed that there were 85726, 92914, and 100421 differentially methylated CpGs and 5940, 14804, and 16893 differentially methylated CpHs (where H = C, T, or A) in the hypothalamic methylomes. The methylation changes of CpHs were more dynamic than that of CpGs, and methylation changes of CpGs and CpHs were likely to be, respectively, involved in the developmental processes of reproduction and the molecular processes of cellular communications in the hypothalamus. Moreover, methylation changes of CpHs were observed to overrepresent in the quantitative trait loci of age at puberty, and the biological function of these CpH methylation changes was enriched in the pancreas development in gilts. Furthermore, the mRNA levels of several differentially CpG or CpH methylated genes related to the transcription of RNA II polymerase, GnRH signaling pathway, Estrogen signaling pathway, PI3K-AKt signaling pathway, and Insulin signaling pathway, including *MAX*, *MMP2*, *FGF11*, *IGF1R*, *FGF21*, and *GSK3B*, were significantly changed across these pubertal stages in the hypothalamus. These results will help our understanding of how DNA methylation contributes to phenotypic variation of age at puberty.

## Introduction

In female pigs, the onset of puberty is widely thought to be the processes of sexual maturations of gilts to be capable of reproduction. Many evidences suggest that gilts that have an earlier age at puberty are more likely to farrow multiple litters (Tummaruk et al., 2007), give birth to more piglets (Patterson et al., 2010) and have a longer reproductive longevity (Roongsitthichai et al., 2014). Nevertheless, with the intensive artificial selection for growth rate and lean meat, recent studies have suggested that approximately 30% of gilts are culled due to the delayed puberty (Stancic et al., 2009, 2011) with failure to display the first estrus by 240 days (Nonneman et al., 2014), which is consequent in a heavily economic burden on the modern commercial farms. However, the underlying molecular mechanism regarding the regulation of the onset of puberty has remained largely unexplored in pigs.

In gilts, age at puberty, a trait with moderate heritability (*h*^2^ = 0.38 to 0.46) (Kuehn et al., 2009), is usually defined as the day of first standing estrus in modern commercial farms. Using 759 gilts genotyped by Illumina PorcineSNP 60 BeadChip, Nonneman et al. (2016) found two quantitative trait loci (QTLs) which explained 16.87% of the total genetic variance of age at puberty. Using the imputed whole genome sequences, Xin et al. (2018) identified 2339 SNPs which were associated with pubertal reproductive failure, explaining 16% of the phenotypic variances. These studies suggest that genetic factors only can explain and account for ∼16% of the phenotypic variances of age at puberty in pigs. Recently, changes of DNA methylation induced by 5-azacytidine have been demonstrated to disrupt the pulsatile release of gonadotropin-releasing hormone (GnRH) from the hypothalamus and delay the onset of puberty (Lomniczi et al., 2013, 2015), suggesting that DNA methylation plays a vitally regulatory role in the onset of puberty and may contribute to the phenotypic variation of age at puberty in pigs.

In female pigs, the onset of puberty is regulated and driven by GnRH released from the hypothalamus (Pelletier et al., 1981; Trout et al., 1984). We hypothesized that DNA methylation of hypothalamus might exhibit dramatic changes across the pubertal transition in gilts. Herein, the objectives of this study were to profile the genome-scale DNA methylation of hypothalamus for Pre-, In- and Post-pubertal gilts by using the reduced representation bisulfite sequencing (RRBS), and these methylation profiles were compared to describe the dynamics and changes across pubertal transition in both CpG and CpH (where *H* = C, T, or A) contexts. Then the association between methylation changes and QTLs of age at puberty in pigs were further explored, and the biological functions of these methylation changes were identified during the onset of puberty. These results are expected to provide useful insights into the epigenetic mechanism for the onset of puberty in gilts.

## Materials and Methods

### Ethics Statement

The pig cares and the experiments were conducted according to the Regulations for the Administration of Affairs Concerning Experimental Animals (Ministry of Science and Technology, China) and were approved by the Animal care and Use Committer of South China Agricultural University, Guangzhou, China (approval number: SCAU#2013-10). The pigs were fed the same diet *ad libitum* and reared under the same conditions in the same environments.

### Animals and Sample Preparation

The hypothalamus samples were collected from Landrace × Yorkshire crossbred gilts. The onset of pig puberty could be handily identified by the standing reflex with the back-pressure test and boar contact (Patterson et al., 2002). 25 Landrace × Yorkshire crossbred gilts aged at 160 days were selected and used in this study. Pubertal signs were checked and recorded twice daily at 09:00 and 15:30 by inspection of the vulva and assessment of the standing reflex for these 25 gilts. Three gilts aged 180 days without pubertal signs were selected as the Pre-pubertal gilts; three gilts at the day exhibiting the first estrus and standing reflex were selected as the In-pubertal gilts (about 205 days); and another three gilts in the dioestrus phase, 14 days after the day exhibiting the first estrus and standing reflex, were selected as the Post-pubertal gilts. Pigs were fed the same diet daily and reared in the same conditions and environments. The hypothalamus of each pubertal group was collected from and was frozen quickly in liquid nitrogen and then stored at −80°C for further using.

### Constructions of RRBS Library

The library constructions and sequencing services were provided by RiboBio Co., Ltd., (Guangzhou, China) as previously described in our studies (Yuan et al., 2016, 2017a,b). The genomic DNA of these hypothalamus tissues was extracted using a DNeasy Blood and Tissue Kit (Qiagen, Beijing), and then, after checking on the quality of the extracted DNA, one library was built for each gilts based on previously published RRBS studies (Yuan et al., 2016, 2017a,b). The processes and procedures of RRBS libraries were briefed as follows. Firstly, the purified genomic DNA was digested overnight with *Msp*I (New England Biolabs, United States). For the *Msp*I digested segments, the sticky ends were filled with CG nucleotides and 3′ A overhangs were added. Secondly, methylated Illumina sequencing adapters with 3′ T overhangs were ligated to the digested segments, and the products obtained were purified. Then 110–220 bp fragments were selected (Yuan et al., 2017b) and converted by bisulfite using an EZ DNA Methylation Gold Kit (Zymo Research, United States). Lastly, libraries of 110–220 bp fragments were PCR amplified by 18 cycles, and each library was sequenced using one lane of an Illumina HiSeq 2500 and 100 bp paired-end reads.

### Bioinformatic Analysis of RRBS Data

The first two nucleotides were trimmed from all the second read sequences to blunt-end the *Msp*I site. All reads were trimmed using Trim Galore (v0.4.0) software (Babraham Bioinformatics^1^) and a Phred quality score of 20 as the minimum. The adaptor pollution reads and multiple N reads (where *N* > 10% of one read) were removed to generate the clean reads. The quality control checks were performed by FastQC (v0.11.3) software (Babraham Bioinformatics). The clean reads were analyzed and performed by Bismark (v0.14.5) (Krueger and Andrews, 2011) using the default parameters. The clean reads were mapped to the pig reference genome (Sscrofa 11.1, downloaded from Ensembl^2^), by using “bismark” function, the PCR duplicates were removed by using “deduplicate_bismark” function, and then the DNA methylation calling was extracted by using “bismark_methylation_extraxtor” function.

After DNA methylation calling by Bismark for these nine RRBS data, 1320853 CpGs and 5884256 CpHs covered by at least five reads and coexisted across all tissues were remained for further analysis. The methylation level of the CpGs was calculated as the methylated reads divided the total covered reads. The average methylation level of each hypothalamic group was calculated by the average methylation level across the three replicates. For each specific region, the methylation level was measured as the average level of CpGs located in this region. To profile the DNA methylation patterns at the gene and CGI locations, the gene locations were divided into 20, 40, and 20 bins for 5 kb upstream region of the transcription start sites (TSSs), gene body and 5 kb downstream region of transcription end sites (TESs), respectively, and the CGI locations were divided into 20, 20, and 20 bins for 2 kb upstream region, CGIs and 2 kb downstream region, respectively. These analyses were performed by Perl and R scripts. This pipeline was carefully described by our previous studies (Yuan et al., 2016, 2017a).

### Annotation of Detected CpGs and CpHs

The locations of porcine CGIs were downloaded from UCSC^3^. CGIs were denoted as the same to UCSC and described as regions >200 bp in length, with a C and G percentage >0.5, and a ratio of the observed CpG/expected CpG > 0.6. The expected CpG was calculated as the number of Cs multiplied by the number of Gs, divided by the length of the segment. The +/−2 kb regions outside of CGIs were defined as CGI shores, and the +/−2 kb regions outside of CGI shores were defined as CGI shelves.

The locations of genes were downloaded from Ensembl (see text footnote 2). Based on genic locations of Ensembl, the porcine genome was separated into five genic features, which were upstream, exonic, intronic, downstream and intergenic regions. The upstream region was 5 kb upstream region of the TSS. The exon was defined as the integration of 5′UTR, CDS, and 3′UTR arranging from the TSS to the TES. The intron was determined as the integration of introns arranged from the TSS to the TES. The downstream region was 5 kb downstream region of the TES. The intergenic region was denoted as the outside regions of upstream, exonic, intronic, and downstream regions.

### Quantitative Real-Time PCR

The total RNA was extracted using TRIzol reagent (TaKaRa, Tokyo, Japan) and reverse-transcribed using a RevertAid First Strand cDNA Synthesis Kit (Thermo Fisher Scientific, United States) for mRNAs. The relative expression levels of the mRNAs were quantified using Maxima SYBR Green qRT-PCR Master Mix (2×) (Thermo Fisher Scientific) and THUNDERBIRD SYBR qPCR Mix (Toyobo) on a LightCycler Real-Time PCR system. The expression levels of *GAPDH* mRNAs were used as endogenous controls, and the fold changes were calculated using the 2^−ΔΔct^ method. The primer sequences are listed in Supplementary Table S1.

### Statistics Analysis

The significant differences between two groups were tested using the Student’s test. The CGIs were identified as the differentially methylated CGIs (DMIs) if the average methylation levels of CGIs, which were covered with at least 20 CpGs or CpHs, were significantly different according to the *P*-value from the Student’s test corrected by the false discovery rate (*P* ≤ 0.05). The genes, including 5 kb upstream flanking region, gene body and 5 kb downstream flanking region, were identified as the differentially methylated genes (DMGs) if the average methylation levels of genes, which were covered with at least 20 CpGs or CpHs, were significantly different according to the *P*-value from the Student’s test corrected by the false discovery rate (*P* ≤ 0.05). The genes, including 5 kb upstream flanking region, gene body and 5 kb downstream flanking region, overlapped at least one DMIs were defined as the DMIs regarding genes.

The differentially methylated CpG (DMCs) and CpH sites (DMHs) were calculated by CGmapTools (Guo et al., 2018). The CpGs or CpHs, whose methylation levels changed more than 20%, were identified as DMCs or DMHs according to the *P*-value from a two-tail Fisher’s exact test corrected by the false discovery rate (*P* ≤ 0.05). For the comparison between two groups, CpGs and CpHs with higher methylation level in one group were considered as the hyper-methylated CpGs or CpHs in this group and were considered as the hypo-methylated CpGs or CpHs in another group, respectively. The genes, including 5 kb upstream flanking region, gene body and 5 kb downstream flanking region, contained at least one DMC were defined as the DMC regarding genes. The genes, including 5 kb upstream flanking region, gene body and 5 kb downstream flanking region, contained at least one DMH were defined as the DMH regarding genes. The enrichment for certain genomic regions was calculated by using a two-tail Fisher’s exact test. The Gene Ontology (GO) and Kyoto Encyclopedia of Gene and Genomes (KEGG) enrichment analysis were undertaken by R package “clusterProfiler” (Yu et al., 2012).

## Results

### Genome-Wide DNA Methylation of Hypothalamus During the Onset of Puberty

**FIGURE 1:**
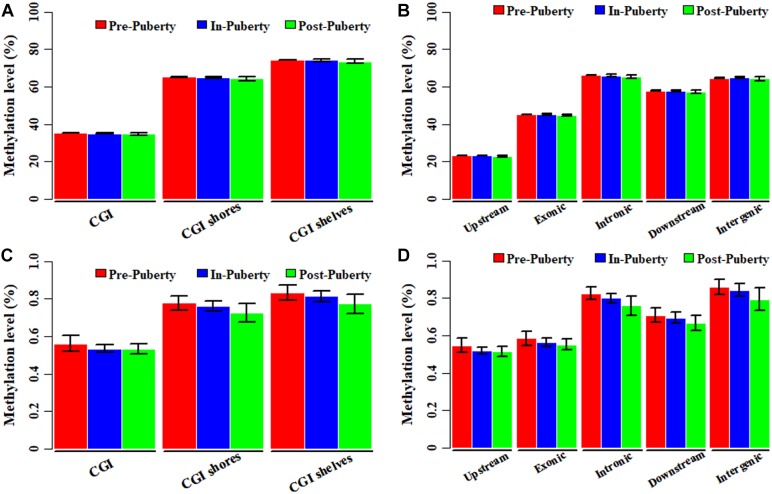
Genome-wide DNA methylation of hypothalamus tissues across pubertal transition in gilts. Average methylation levels of CpGs in CGI- **(A)** and gene-related regions **(B)**. Average methylation levels of CpHs in CGI- **(C)** and gene-related regions **(D)**.

**FIGURE 2:**
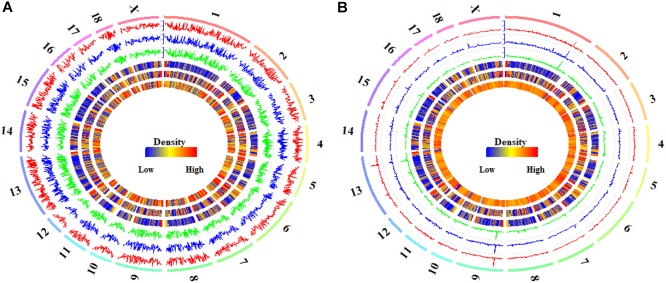
Global DNA methylation of CpGs and CpH in hypothalamus tissues. The global CpG **(A)** and CpH **(B)** methylation levels in Pre- (track 1), In- (track 2), and Post-pubertal (track 3) methylomes of hypothalamus tissues, from outside to inside, were quantified per 1 Mb window. The densities of CGIs (track 4), genes (track 5) and CpGs (track 6 in **A**) or CpHs (track 6 in **B**) were also quantified per 1 Mb window. The labels outside of track 1 represent the chromosomes of the porcine genome.

In the CpG context, the average methylation levels were 53.57% ± 0.07, 53.50% ± 0.41, and 53.01% ± 0.83% for Pre-, In-, and Post-puberty, respectively. The average methylation levels of CGI-related (Figure 1A) and gene-related regions (Figure 1B) were the highest in Pre- and the lowest in Post-pubertal methylome. Compared to other CGI- or gene-related regions (Figure 1A,B), the average methylation levels of CGIs and upstream regions displayed the lowest levels. In the CpH context, the average methylation levels were 0.75% ± 0.04, 0.73% ± 0.03, and 0.70% ± 0.05% for Pre-, In-, and Post-puberty, respectively. The CpH methylation levels of CGI-related (Figure 1C) and gene-related regions (Figure 1D) displayed the same pattern with that in CpG methylomes (Figure 1A,B). For Pre- vs. In-, In- vs. Post- and Pre- vs. Post-puberty, the Pearson’s correlation coefficients of methylation levels were all 0.99 (*P* < 2.22 × 10^−16^) (Figure 2A and Supplementary Table S2) for CpGs but were 0.82 (*P* < 2.22 × 10^−16^), 0.85 (*P* < 2.22 × 10^−16^), and 0.89 (*P* < 2.22 × 10^−16^) for CpHs (Figure 2B and Supplementary Table S2), respectively.

### DNA Methylation Patterns of CpGs and CpHs in CGI and Gene Locations

**FIGURE 3:**
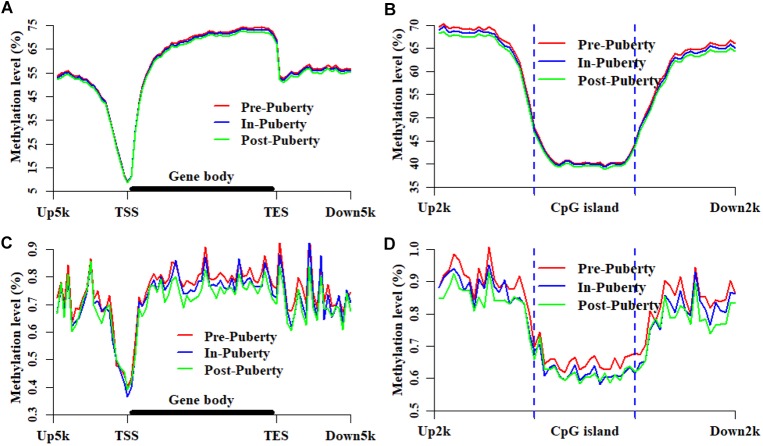
Methylation patterns of CpGs and CpHs at CGI and gene locations. Methylation levels and patterns of CpGs at the locations of genes **(A)** and CGIs **(B)**. Methylation levels and patterns of CpHs at the locations of genes **(C)** and CGIs **(D)**. Up5k is denoted as the 5 kb upstream regions of transcription start sites. Down5k is denoted as the 5 kb downstream regions of transcription end sites. Up2k and Down2k are identified as the 2 kb upstream and downstream regions of the CGIs, respectively.

To explore the dynamics and similarities of DNA methylation at the locations of genes and CGIs during the onset of puberty, the methylation patterns of CpGs and CpHs were profiled for the hypothalamic methylomes of Pre-, In-, and Post-puberty (Figure 3), respectively. We found that the methylation levels of CpGs were likely to overlap together at the locations of genes (Figure 3A) and CGIs (Figure 3B). However, the methylation levels could be clearly distinguished from each other in the upstream or downstream regions around the CGIs (Figure 3B). Besides, the methylated patterns of CpHs (Figure 3C,D) exhibited the similarly methylated patterns with that of CpGs (Figure 3A,B).

To further determine the DNA methylation dynamics at the locations of genes and CGIs, the DMIs and DMGs were calculated for Pre- vs. In-, In- vs. Post- and Pre- vs. Post-puberty, respectively. There were 221, 207, and 236 DMIs, and there were 290, 238, and 281 DMGs in the CpG context (Supplementary Figures S1A–C). There were 538, 568, and 571 DMIs, and there were 329, 300, and 321 DMGs in the CpH context for Pre vs. In-, In- vs. Post- and Pre- vs. Post-puberty (Supplementary Figures S1A–C), respectively. The GO and KEGG pathway analysis were performed on these DMGs and DMIs regarding genes. In the CpG context, the most significantly enriched GO and KEGG terms were Metanephric mesenchyme development, Kidney mesenchyme development, Signaling pathways regulating pluripotency of stem cells, mTOR signaling pathway, Wnt signaling pathway for DMIs regarding genes (Supplementary File S1); and were cardiovascular system development, and Signaling pathways regulating pluripotency of stem cells for DMGs (Supplementary File S1); In the CpH context, the most significantly enriched GO and KEGG terms in CpH were RNA polymerase II transcription factor binding, transcription factor binding, PI3K-Akt signaling pathway, MAPK signaling pathway and Oocyte meiosis for DMIs regarding genes (Supplementary File S2); and were phosphatidylinositol bisphosphate binding, phosphatidylinositol phosphate binding, and Lysine degradation for DMGs (Supplementary File S2).

### Methylation Changes During the Onset of Puberty in Hypothalamic Genome

**FIGURE 4:**
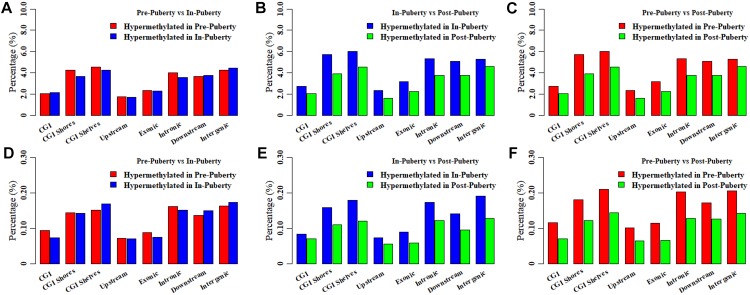
Differentially methylated CpGs and CpHs in hypothalamic methylomes across pubertal transition. Distributions of differentially methylated CpGs between Pre- and In- **(A)**, In- and Post- **(B)**, and Pre- and Post-puberty **(C)**. Distributions of differentially methylated CpHs between Pre- and In- **(D)**, In- and Post- **(E)**, and Pre- and Post-puberty **(F)**.

To investigate the methylation changes among hypothalamic methylomes across the Pre-, In-, and Post-puberty, the DMCs and DMHs were counted for Pre- vs. In-, In- vs. Post- and Pre- vs. Post-puberty. Respectively, 85726, 92914, and 100421 DMCs were identified for Pre- vs. In- (Figure 4A), In- vs. Post- (Figure 4B) and Pre- vs. Post-puberty (Figure 4C), representing 6.49, 7.03, and 7.60% of all detected CpGs (Table 1). These DMCs were observed to be hypo-methylated in Post-methylome but hyper-methylated in Pre- and In-methylomes (Figure 4A–C). Moreover, these DMCs were observed to overrepresent in CGI shores, CGI shelves, Introns, downstream and intergenic regions (relative enrichment = 1.11–1.49, *P* ≤ 9.29 × 10^−3^) (Table 1) but underrepresent in CGI, exons and upstream regions (relative enrichment = 0.46–0.68, *P* < 2.22 × 10^−16^) (Table 1).

**Table 1:** Distribution of differentially methylated CpGs among these hypothalamic methylomes.

	Detected CpGs	DMCs between Pre- and In-		DMCs between In- and Post-		DMCs between Pre- and Post-	
		Number	Enrichment	Number	Enrichment	Number	Enrichment
Total	1320853	85726	—	92914	—	100421	—
CGI	628028	26422	0.49 (*P* < 2.22 × 10^−16^)	28834	0.50 (*P* < 2.22 × 10^−16^)	29862	0.47 (*P* < 2.22 × 10^−16^)
CGI shores	259488	20572	1.29 (*P* < 2.22 × 10^−16^)	22559	1.31 (*P* < 2.22 × 10^−16^)	25014	1.36 (*P* < 2.22 × 10^−16^)
CGI shelves	86548	7621	1.39 (*P* < 2.22 × 10^−16^)	8489	1.43 (*P* < 2.22 × 10^−16^)	9131	1.43 (*P* < 2.22 × 10^−16^)
Upstream	220380	7557	0.48 (*P* < 2.22 × 10^−16^)	7797	0.46 (*P* < 2.22 × 10^−16^)	8716	0.47 (*P* < 2.22 × 10^−16^)
Exon	290064	13384	0.66 (*P* < 2.22 × 10^−16^)	14895	0.68 (*P* < 2.22 × 10^−16^)	15729	0.66 (*P* < 2.22 × 10^−16^)
Intron	419870	31840	1.28 (*P* < 2.22 × 10^−16^)	35490	1.33 (*P* < 2.22 × 10^−16^)	38123	1.31 (*P* < 2.22 × 10^−16^)
Downstream	75956	5644	1.15 (*P* = 9.29 × 10^−3^)	5912	1.11 (*P* = 2.48 × 10^−14^)	6714	1.17 (*P* < 2.22 × 10^−16^)
Intergenic	314583	27301	1.49 (*P* = 2.62 × 10^−4^)	28820	1.44 (*P* < 2.22 × 10^−16^)	31139	1.44 (*P* < 2.22 × 10^−16^)
QTL	57 332	3930	1.06 (*P* = 5.76 × 10^−4^)	4387	1.09 (*P* = 2.30 × 10^−8^)	4691	1.08 (*P* = 4.12 × 10^−7^)

*Enrichment of differentially methylated CpHs for certain genomic regions was calculated by using with a two-tail Fisher’s exact test.*

Then 15940, 14804, and 16893 DMHs were identified for Pre- vs. In- (Figure 4D), In- vs. Post- (Figure 4E), and Pre- vs. Post-puberty (Figure 4F), representing 0.27, 0.25, and 0.29% of all detected CpHs (Table 2), respectively. These DMHs were also observed to be hypo-methylated in Post-methylome but hyper-methylated in Pre- and In-methylomes (Figure 4D–F). Furthermore, we found that DMHs showed a significant underrepresentation in CGIs, upstream regions and exons (relative enrichment = 0.48–0.58, *P* < 2.22 × 10^−16^) (Table 2), but CGI shores, CGI shelves, introns and intergenic regions showed a significant overrepresentation of DMHs (relative enrichment = 1.07–1.41, *P* ≤ 6.53 × 10^−3^) (Table 2). Interestingly, DMHs of downstream regions enriched in a stage-specific manner among the comparisons of Pre- vs. In-, In- vs. Post-, and Pre- vs. Post-puberty (Table 2).

**Table 2:** Distribution of differentially methylated CpHs among these hypothalamic methylomes.

	Detected CpHs	DMHs between Pre- and In-		DMHs between In- and Post-		DMHs between Pre- and Post-	
		Number	Enrichment	Number	Enrichment	Number	Enrichment
Total	5884256	15940	—	14804	—	16893	—
CGI	1611528	2686	0.54 (*P* < 2.22 × 10^−16^)	2456	0.53 (*P* < 2.22 × 10^−16^)	2992	0.57 (*P* < 2.22 × 10^−16^)
CGI shores	1528834	4378	1.08 (*P* = 2.22 × 10^−5^)	4102	1.09 (*P* = 2.05 × 10^−6^)	4620	1.07 (*P* = 5.73 × 10^−5^)
CGI shelves	507135	1620	1.20 (*P* = 1.28 × 10^−11^)	1518	1.21 (*P* = 5.41 × 10^−12^)	1788	1.25 (*P* = 6.53 × 10^−3^)
Upstream	773667	1099	0.50 (*P* < 2.22 × 10^−16^)	997	0.48 (*P* < 2.22 × 10^−16^)	1281	0.54 (*P* < 2.22 × 10^−16^)
Exon	944488	1538	0.56 (*P* < 2.22 × 10^−16^)	1387	0.54 (*P* < 2.22 × 10^−16^)	1690	0.58 (*P* < 2.22 × 10^−16^)
Intron	2130322	6661	1.26 (*P* < 2.22 × 10^−16^)	6251	1.29 (*P* < 2.22 × 10^−16^)	7034	1.26 (*P* < 2.22 × 10^−16^)
Downstream	371205	1063	1.06 (*P* = 6.30 × 10^−2^)	876	0.93 (*P* = 5.16 × 10^−2^)	1102	1.04 (*P* = 2.54 × 10^−1^)
Intergenic	1664574	5579	1.36 (*P* < 2.22 × 10^−16^)	5293	1.41 (*P* < 2.22 × 10^−16^)	5786	1.32 (*P* < 2.22 × 10^−16^)
QTL	251 222	796	1.17 (*P* = 2.435 × 10^−5^)	788	1.25 (*P* = 4.84 × 10^−9^)	826	1.15 (*P* = 1.83 × 10^−4^)

*Enrichment of differentially methylated CpHs for certain genomic regions was calculated by using with a two-tail Fisher’s exact test.*

### Global Distributions of DMCs and DMHs in Hypothalamic Genomes

**FIGURE 5:**
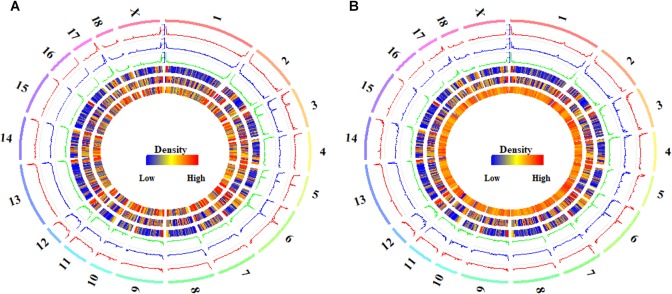
Global distribution of differentially methylated CpG and CpH sites in hypothalamic genomes during the pubertal transition. The global distributions of differentially methylated CpG **(A)** and CpH **(B)** sites in Pre- vs. In- (track 1), In- vs. Post- (track 2), and Pre- vs. Post-pubertal (track 3) methylomes of hypothalamus tissues, from outside to inside, were quantified per 1 Mb window. The densities of CGIs (track 4), genes (track 5), and CpGs (track 6 in **A**) or CpHs (track 6 in **B**) were also quantified per 1 Mb window. The labels outside of track 1 represent the chromosomes of the porcine genome.

The distributions of DMCs and DMHs in the hypothalamic methylomes were profiled in Figure 5. We found that these DMCs and DMHs were likely to enrich at the ends of each chromosome (Figure 5). Whereas DMCs exhibited additional higher peaks in the bodies of chromosome 3, chromosome 5, and chromosome 6 (Figure 5A), and DMHs displayed additional higher peaks in the body of chromosome 6 (Figure 5B). Moreover, DMHs were likely to show a stage-specific distribution among the onset of puberty, e.g., the distributions of DMHs in the chromosome 3 (Figure 5B).

The Pearson’s correlation coefficients among the distributions of DMCs were all 0.99 (*P* < 2.22 × 10^−16^) for Pre- vs. In-, In- vs. Post-, and Pre- vs. Post-puberty (Supplementary Table S3). The distributions of DMCs were observed to be highly positively correlated with the densities of CpGs (Pearson’s correlation coefficients = 0.86, *P* < 2.22 × 10^−16^) and CGIs (Pearson’s correlation coefficients = 0.94, *P* < 2.22 × 10^−16^) (Supplementary Table S3), and the distributions of DMCs were moderately linked with the densities of genes (Pearson’s correlation coefficients = 0.32, *P* < 2.22 × 10^−16^) (Supplementary Table S3). However, the Pearson’s correlation coefficients among the distributions of DMHs were 0.94 (*P* < 2.22 × 10^−16^), 0.93 (*P* < 2.22 × 10^−16^), and 0.95 (*P* < 2.22 × 10^−16^) for Pre- vs. In-, In- vs. Post-, and Pre- vs. Post-puberty (Supplementary Table S3), respectively. The distributions of DMHs were highly associated with the densities of CGIs (Pearson’s correlation coefficients = 0.85–0.86, *P* < 2.22 × 10^−16^) and CpHs (Pearson’s correlation coefficients = 0.54–0.57, *P* < 2.22 × 10^−16^) but were slightly correlated with the densities of genes in hypothalamic methylome (Pearson’s correlation coefficients = 0.16–0.18, *P* ≤ 3.94 × 10^−10^) (Supplementary Table S3).

### Enrichments of DMCs and DMHs in the QTL of Age at Puberty in Pigs

To further explore the associations between DNA methylation changes and the QTLs of age at puberty in pigs, 237 QTLs of age at puberty were collected from PigQTLdb (Release 36). These QTLs covered 57332 CpGs and 251222 CpHs, respectively. The numbers of DMCs located at these QTLs were 3930, 4387, and 4691 for Pre- vs. In-, In- vs. Post-, and Pre- vs. Post-puberty, respectively (Table 1). Compared to the whole hypothalamic genome, these DMCs were not obviously enriched in QTLs (relative enrichment = 1.06–1.09, *P* ≤ 5.76 × 10^−4^) (Table 1). The number of DMHs located at QTLs were 796, 788, and 826 for Pre- vs. In-, In- vs. Post-, and Pre- vs. Post-puberty, respectively (Table 2). Moreover, these DMHs were observed to overrepresent in QTLs (relative enrichment = 1.15–1.25, *P* ≤ 1.83 × 10^−4^) (Table 2). To gain insight into the biological functions in which methylation changes of CpHs might be involved, we performed the biological processes of GO and KEGG pathway analysis on genes (Supplementary File S3) that associated with at least one DMH located at QTLs. We found no signaling pathway was enriched, and only one GO term, pancreas development, was enriched.

### Biological Functions of DMCs and DMHs in Hypothalamic Genomes Across the Pubertal Transition

**FIGURE 6:**
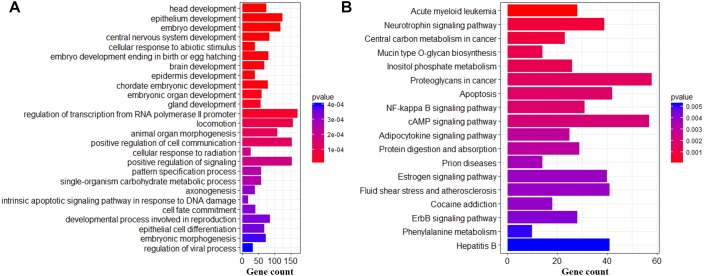
Biological functions of DMCs in hypothalamic genomes across the pubertal transition. Significantly enriched GO terms **(A)** and KEGG pathways **(B)** of DMC genes.

**FIGURE 7:**
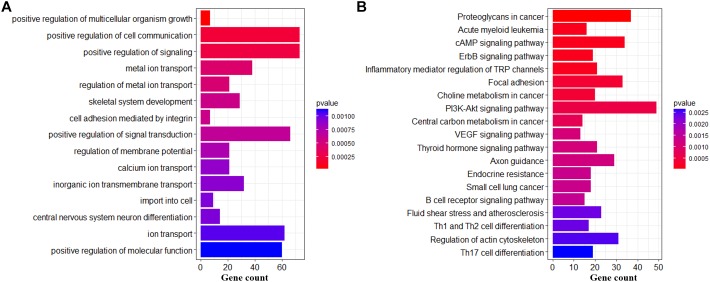
Biological functions of DMHs in hypothalamic genomes across the pubertal transition. Significantly enriched GO terms **(A)** and KEGG pathways **(B)** of DMH genes.

To further gain insight into the biological functions in which DMC or DMH genes might be involved, we performed the biological processes of GO and KEGG pathway analysis on genes who were associated with at least one DMC (Figure 6) and DMH (Figure 7). For DMCs regarding genes (Supplementary File S4), we found that the most significantly enriched GO terms were the development (head development, central nervous system development, brain development, gland development, etc.), developmental process involved in reproduction, regulation of transcription from RNA polymerase II promoter, animal organ morphogenesis, and so on (Figure 6A). Moreover, these genes were significantly enriched in Acute myeloid leukemia, Neurotrophin signaling pathway, Apoptosis, cAMP signaling pathway, Estrogen signaling pathway, and so on (Figure 6B).

For DMHs regarding genes (Supplementary File S4), the most significantly enriched terms were the ion transport (metal ion transport, regulation of metal ion transport, calcium ion transport, inorganic ion transmembrane transport, etc.), positive regulation of cell communication, positive regulation of signaling, import into cell, central nervous system neuron differentiation and so on (Figure 7A). Moreover, these genes were significantly enriched in PI3K-AKt signaling pathway, Proteoglycans in cancer, Focal adhesion, Axon guidance, Acute myeloid leukemia, Regulation of actin cytoskeleton, and so on (Figure 7B).

**FIGURE 8:**
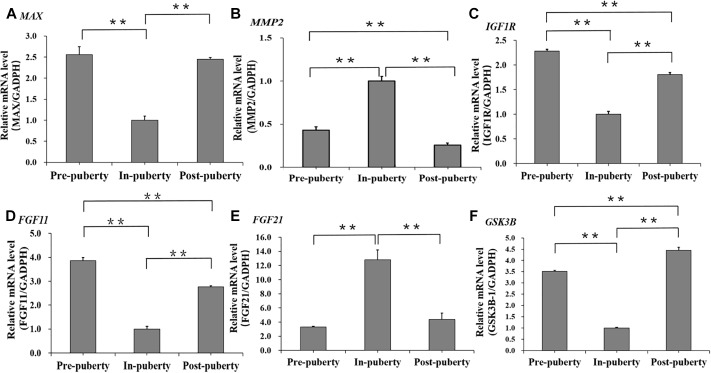
mRNA expression levels of several DMC or DMH regarding genes across the pubertal transition in hypothalamus. *MAX* **(A)**, *MMP2* **(B)**, and *FGF11* **(C)** were DMC regarding genes. *IGF1R* **(D)**, *FGF21* **(E)**, and *GSK3B* **(F)** were DMH regarding genes. The significance of differences in means between two groups was analyzed by using Student’s *t*-test (two-tailed). Data were expressed as means ± standard error of biological replicates. ^∗∗^ indicates *P* < 0.01.

Furthermore, the mRNA expressions of several DMC or DMH regarding genes selected basing on literatures were detected across the pubertal transition in hypothalamus. We found that the mRNA levels of *MAX* (Figure 8A), *MMP2* (Figure 8B), and *FGF11* (Figure 8C), which were the DMC regarding genes and associated with the transcription of RNA II polymerase, GnRH signaling pathway and Estrogen signaling pathway, were significantly changed across Pre-, In-, and Post-puberty in hypothalamus. Also, the mRNA levels of *IGF1R* (Figure 8D), *FGF21* (Figure 8E) and *GSK3B* (Figure 8F), which were DMH regarding genes and associated with PI3K-AKt signaling pathway and Insulin signaling pathway, were significantly changed across Pre-, In-, and Post-puberty in hypothalamus.

## Discussion

Although the early puberty is suggested to improve the reproductive performance and longevity (Patterson et al., 2010; Roongsitthichai et al., 2014), collecting age-at-puberty data for selection of the early puberty is tedious and labor intensive, which requires daily estrus detection in the presence of a boar. The appearance that approximately 30% of gilts are culled due to the delayed puberty (Stancic et al., 2009, 2011) markedly harms the financial stake of the modern commercial farms. Recent advance made in “omics” technologies are allowed for exploring the complicated mechanism underlying the onset of puberty and contributing to the selection and breeding practices of age at puberty in gilts.

In this study, we found that the methylation levels of CpGs were likely to overlap together at the genic regions (Figure 3A) and CGIs (Figure 3B). However, the methylation levels of CpHs were likely to be clearly distinguished from each other at gene bodies (Figure 3C) and at the locations of and CGIs (Figure 3D). Respectively, the Pearson’s correlation coefficients were all 0.99 (*P* < 2.22 × 10^−16^) (Figure 2A and Supplementary Table S2) for the methylation levels of CpGs but were 0.82–0.89 (*P* < 2.22 × 10^−16^) (Figure 2B and Supplementary Table S2) for the methylation levels of CpGs between Pre- vs. In-, In- vs. Post-, and Pre- vs. Post-puberty. Furthermore, during the pubertal transition, the numbers of DMIs and DMGs in the CpG context were more than that in the CpH context (Supplementary Figures S1A–C). These results indicated that the methylation levels of CpHs were more variable than that of CpGs among the hypothalamic methylomes of Pre-, In-, and Post-puberty.

The DNA methylation changes of CpGs were likely to play an important role in regulation of transcription from RNA polymerase II, Neurotrophin signaling pathway and Estrogen signaling pathway (Figure 6B), which were strongly associated with biological functions of hypothalamus for the onset of puberty in pigs (Trout et al., 1984; Ieda et al., 2014). Besides, compared to Pre- and Post-puberty, we found that *MAX*, which was *MYC* associated factor X and might stimulate the transcription of RNA II polymerase (Campbell and White, 2014), showed the lowest mRNA level in In-puberty (Figure 8A). *MMP2*, which was one of the key genes of the GnRH signaling pathway and Estrogen signaling pathway^4^, showed the highest mRNA level in In-puberty (Figure 8B). *IGF1R*, which was a key gene of Ovarian steroidogenesis pathway^5^ and played an important role in controlling the timing of puberty (Chang et al., 2018), showed the lowest mRNA level in In-puberty (Figure 8C). These observations suggested that the CpG methylation changes were likely to show a crucial regulatory role in controlling the transcription of genes related to the onset of puberty at hypothalamus level in gilts.

However, the DNA methylation changes of CpHs were mostly found to get involved in the ion metabolizing and the positive regulation of cell communication, import into cell, neuron differentiation (Figure 7A), which were likely to get involved in the maintenance of molecular function within cellular communications (Jang et al., 2017; Lee et al., 2017). Additionally, compared to Pre- and Post-puberty, *FGF11*, which was one of the key genes of PI3K-AKt signaling pathway, presented the lowest mRNA level in In-puberty (Figure 8D), but *FGF21*, which expressed in GnRH neurons and regulated GnRH secretion during the onset of puberty in mice (Misrahi, 2017), displayed the highest mRNA level in In-puberty (Figure 8E). *GSK3B*, which was one of genes of Insulin signaling pathway^6^ and associated with age of puberty in cattle (Fortes et al., 2013), displayed the lowest mRNA level in In-puberty (Figure 8F). These results indicated that methylation changes of CpGs were involved in the maintenance of molecular function within cellular communications during the initiation of puberty in gilts.

Recently, the genome-wide DNA methylation changes of hypothalamus have been investigated during the timing of puberty in rat (Luo et al., 2017) and goat (Yang et al., 2016), even the methylation variation between rat and goat (Yang et al., 2018). Results of rat (Luo et al., 2017) and goat (Yang et al., 2016) show that the global DNA methylation level is decreasing across Pre- to In-pubertal stages, which is in line with the present study. We found that the average CpG and CpH methylation levels of genomes, GCI- and gene-related regions in hypothalamic genomes were gradually decreased across Pre-, In-, and Post-puberty (Figure 1). It seems that the decreasing trend of global DNA methylation level from Pre- to In-pubertal stage happens whatever the mammalian species is, although the difference is not statistically significant. This decrease of the global DNA methylation, from Pre- to In-pubertal stage, may be related to the DNA methylation changes of genes which represses the initiating of puberty in Pre-puberty, and these genes may change from transcriptional repressions to activations. Moreover, many DMGs such as NLRC5, SMOC1, GRID1, ABAT, MAP3K4, and *PTPRN* reported in rat (Yang et al., 2018) and goat (Yang et al., 2018) across the pubertal transition were also identified in this study (Supplementary File S4). Furthermore, changes of the DNA methylation in the hypothalamus were all enriched in Neurotrophin signaling pathway and Estrogen signaling pathway for rat (Yang et al., 2018), goat (Yang et al., 2018)and pigs (Figure 6). These observations not only supported the accuracies of findings in the present study but also suggested the patterns of DNA methylation variations were like to be similar among goat, rat and pigs.

With the intensive artificial selection for growth rate and lean meat, recent studies have suggested that approximately 30% of gilts are culled due to the delayed puberty (Stancic et al., 2009, 2011) with failure to display the first estrus by 240 days (Nonneman et al., 2014). We found that, compared to DMCs (Table 1), DMHs were observed to overrepresent in QTLs of age at puberty in gilts (Table 1), and genes, which were associated with at least one DMH located at QTLs of age at puberty, were only significantly enriched in the GO term of pancreas development, which is highly associated with the onset of puberty in mammals (Bervoets et al., 2015). Previous studies have shown that the CpH methylation may play a repressive role in the gene’s transcription (Luo et al., 2016; Keown et al., 2017), and Insulin secreted from pancreases promotes the synthesis of glycogen, fat and protein (Bervoets et al., 2015; Singh et al., 2017), and the gilts with lean body composition were likely to show the delayed puberty (Gaughan et al., 1997). Moreover, compared to Pre- and Post-pubertal stages, *IGF1R* (Figure 8C) and *GSK3B* (Figure 8F), which are highly involved in synthesis and secretion of Insulin, show the lowest mRNA levels at In-pubertal stage. It is possible that the intensive artificial selection for growth rate and lean meat has damaged the development of pancreas and secretion of Insulin and then impacts the body composition and the timing of puberty in gilts. These observations recommend that DNA methylation may contribute to the phenotypic variation of age at puberty in pigs.

## Conclusion

Collectively, during the pubertal transition in gilts, the methylation changes of CpHs were more dynamic than that of CpGs, and methylation changes of CpGs and CpHs were likely to be, respectively, involved in the developmental processes of reproduction and the molecular processes within cellular communications in the hypothalamus. Moreover, methylation changes of CpHs were observed to overrepresent in the QTLs of age at puberty, and the biological function of these CpG methylation changes were enriched in the pancreas development and might impact the body composition and the timing of puberty of gilts. These DNA methylation analyses will help our understanding of how DNA methylation contributes to phenotypic variation of age at puberty.

## Data Availability

Publicly available datasets were analyzed in this study. This data can be found here: http://www.ebi.ac.uk/ena/data/view/PRJEB31293.

## Author Contributions

XY, HZ, and JL conceived and designed the work. XY, XZ, YH, SY, and ZC acquired the biological samples and analyzed the data. XY, XZ, YK, and JL drafted the manuscript. NG, HZ, ZZ, and JL critically revised the manuscript. All authors reviewed and approved the final manuscript.

## Conflict of Interest Statement

The authors declare that the research was conducted in the absence of any commercial or financial relationships that could be construed as a potential conflict of interest.

## Footnotes

1 http://www.bioinformatics.babraham.ac.uk/projects/trim_galore/

2 http://asia.ensembl.org/Sus_scrofa/Info/Index

3 http://hgdownload.soe.ucsc.edu/goldenPath/susScr11/database/

4 https://www.genome.jp/dbget-bin/www_bget?hsa:4313+hsa:4318

5 https://www.genome.jp/kegg-bin/show_pathway?hsa04913+3480

6 https://www.genome.jp/kegg-bin/show_pathway?ko04910+K03083

## Supplementary Material

FIGURE S1 Numbers of differentially methylated CpG islands and genes in hypothalamic methylomes across pubertal transition. The number of differentially methylated CpG islands (DMIs) and genes (DMGs) in Pre- vs. In-pubertal **(A)**, In- vs. Post-pubertal **(B)**, and Pre- vs. Post-pubertal **(C)** hypothalamus in the CpG or CpH context.

TABLE S1 Primers used in the present study.

TABLE S2 Correlation coefficients of CpG and CpH methylation with gene and CGI densities.

TABLE S3 Correlation coefficients of DMCs and DMHs with gene and CGI densities.

FILE S1 Significantly enriched GO terms and KEGG pathways of DMGs and DMIs regarding genes in the CpG context.

FILE S2 Significantly enriched GO terms and KEGG pathways of DMGs and DMIs regarding genes in the CpH context.

FILE S3 Regarding genes of DMCs and DMHs in QTLs.

FILE S4 Regarding genes of DMCs and DMHs in hypothalamic genomes.
