# Supplementary material for: Genome-Wide DNA Methylation Analysis of Hypothalamus During the Onset of Puberty in Gilts
Source: Front Genet. 2019 Mar 19;10:228. doi: 10.3389/fgene.2019.00228 (PMC6433709; doi:10.3389/fgene.2019.00228)
Supplement: FIGURE S1 — Numbers of differentially methylated CpG islands and genes in hypothalamic methylomes across pubertal transition. The number of differentially methylated CpG islands (DMIs) and genes (DMGs) in Pre- vs. In-pubertal (A), In- vs. Post-pubertal (B), and Pre- vs. Post-pubertal (C) hypothalamus in the CpG or CpH context. [file Data_Sheet_1.ZIP › Supplementary Material.docx]

**Genome-wide DNA methylation analysis of hypothalamus during the onset of puberty in gilts**

**Xiaolong Yuan^#1^, Xiaofeng Zhou^#1^, Zitao Chen^1^, Yingting He^1^, Yaru Kong^1^, Shaopan Ye^1^, Ning Gao^2^, Zhe Zhang^1^, Hao Zhang***^1^ and **Jiaqi Li***^1^

1. National Engineering Research Center for Breeding Swine Industry, Guangdong Provincial Key Lab of Agro-Animal Genomics and Molecular Breeding, College of Animal Science, South China Agricultural University, Guangzhou, Guangdong, China.

2. State Key Laboratory of Biocontrol, School of Life Sciences, Sun Yat-sen University, North Third Road, Guangzhou Higher Education Mega Center, Guangzhou, Guangdong 510006, China

*Corresponding author: Jiaqi Li, E-mail: [jqli@scau.edu.cn](mailto:jqli@scau.edu.cn); Hao Zhang, E-mail: zhanghao[@scau.edu.cn](mailto:@scau.edu.cn);

^#^These authors contributed equally to this work

**1 Supplementary Figures and Tables**

**1.1 Supplementary Figures**


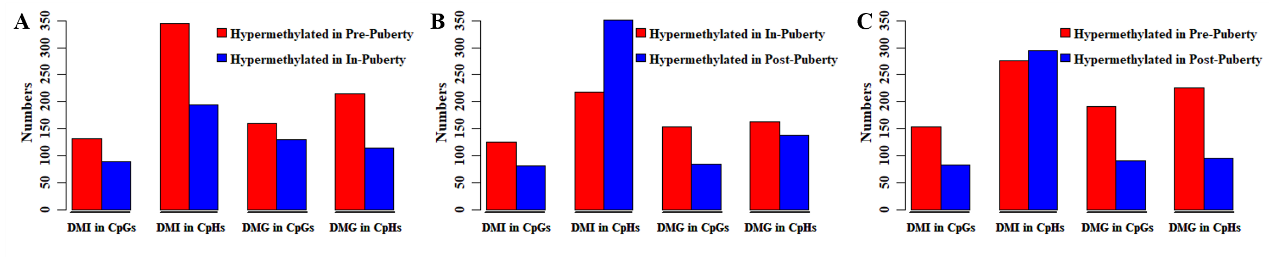


**Supplementary Figure 1. Numbers of differentially methylated CpG islands and genes in hypothalamic methylomes across pubertal transition.** The number of differentially methylated CpG islands (DMIs) and genes (DMGs) in Pre- vs. In-pubertal (**A**), In- vs. Post-pubertal (**B**), and Pre- vs. Post-pubertal (**C**) hypothalamus in the CpG or CpH context.

**1.2 Supplementary Tables**

**Supplementary Table 1. Primers used in the present study**

| Name | Sequence | Product（bp） | Accession number |
| --- | --- | --- | --- |
| *FGF11* | F：GTCACCAAACTGTTCTGCCG  R：GCTTGGCACTCTGAATGGTG | 311 | [XM_013981311.2](https://www.ncbi.nlm.nih.gov/nuccore/XM_013981311.2) |
| *FGF21* | F：CACGAAACTGAAGCCCACCTG  R：TTGTAGCCATCCTCAAGAAGC | 165 | [NM_001163410.1](https://www.ncbi.nlm.nih.gov/nuccore/NM_001163410.1) |
| *GSK3B* | F：AACCACCTTCTTTGCGGAG  R：GCTTGGCTTGATACACGACA | 214 | [NM_001128443.1](https://www.ncbi.nlm.nih.gov/nuccore/NM_001128443.1) |
| *IGF1R* | F：AGAACTGCACGGTGATCGAG  R：AGATGACCAGGGCGTAGTTG | 178 | [NM_214172.1](https://www.ncbi.nlm.nih.gov/nuccore/NM_214172.1) |
| *MAX* | R：ACCAACCTCAAGGACATCGG  F：GTCGTCTTCTCACACAGCGG | 232 | [XM_005666316.3](https://www.ncbi.nlm.nih.gov/nuccore/XM_005666316.3) |
| *MMP2* | R：GATGGCTTCCTTTGGTGTTCC  R：CTTCTTGTCGCTGTCGTAGTCC | 318 | [NM_214192.2](https://www.ncbi.nlm.nih.gov/nuccore/NM_214192.2) |
| *GAPDH* | F：TCCCGCCAACATCAAAT  R：CACGCCCATCACAAACAT | 253 | [NM_001206359.1](https://www.ncbi.nlm.nih.gov/nuccore/NM_001206359.1) |

**Supplementary Table 2. Correlation coefficients of CpG and CpH methylation with gene and CGI densities**

|  | | **Hypothalamic methylomes** | | | **Densities of genes** | **Densities of CGIs** | **Densities of CpHs** |
| --- | --- | --- | --- | --- | --- | --- | --- |
|  |  | **Pre-puberty** | **In-puberty** | **Post-puberty** |  |  |  |
| **Hypothalamic methylomes** | **Pre-puberty** | — | 0.83 (*P* < 2.22 × 10^−16^) | 0.85 (*P* < 2.22 × 10^−16^) | -0.12 (*P* = 1.33 × 10^−8^) | 0.13 (*P* = 2.17 × 10^−9^) | 0.21 (*P* < 2.22 × 10^−16^) |
|  | **In-puberty** | 0.99 (*P* < 2.22 × 10^−16^) | — | 0.89 (*P* < 2.22 × 10^−16^) | -0.11 (*P* = 1.95 × 10^−7^) | 0.12 (*P* = 1.61 × 10^−8^) | 0.23 (*P* < 2.22 × 10^−16^) |
|  | **Post-puberty** | 0.99 (*P* < 2.22 × 10^−16^) | 0.99 (*P* < 2.22 × 10^−16^) | — | -0.11 (*P* = 1.91 × 10^−7^) | 0.14 (*P* = 7.27 × 10^−12^) | 0.22 (*P* < 2.22 × 10^−16^) |
| **Densities of genes** | | -0.11 (*P* = 6.53 × 10^−8^) | -0.12 (*P* = 2.35 × 10^−8^) | -0.12 (*P* = 1.40 × 10^−8^) | — | 0.42 (*P* < 2.22 × 10^−16^) | 0.55 (*P* < 2.22 × 10^−16^) |
| **Densities of CGIs** | | 0.29 (*P* < 2.22 × 10^−16^) | 0.29 (*P* < 2.22 × 10^−16^) | 0.29 (*P* < 2.22 × 10^−16^) | 0.42 (*P* < 2.22 × 10^−16^) | — | 0.60 (*P* < 2.22 × 10^−16^) |
| **Densities of CpGs** | | 0.22 (*P* < 2.22 × 10^−16^) | 0.21 (*P* < 2.22 × 10^−16^) | 0.21 (*P* < 2.22 × 10^−16^) | 0.50 (*P* < 2.22 × 10^−16^) | 0.88 (*P* < 2.22 × 10^−16^) | — |

Correlation coefficients were calculated by Pearson’s correlation. The lower triangle represents the correlation coefficient of CpG methylomes, and the upper triangle represents the correlation coefficient of CpH methylomes.

**Supplementary Table 3. Correlation coefficients of DMCs and DMHs with gene and CGI densities**

|  | | **Hypothalamic methylomes** | | | **Densities of genes** | **Densities of CGIs** | **Densities of CpHs** |
| --- | --- | --- | --- | --- | --- | --- | --- |
|  |  | **Pre- vs. In-puberty** | **In- vs. Post-puberty** | **Pre- vs. Post-puberty** |  |  |  |
| **Hypothalamic methylomes** | **Pre- vs. In-puberty** | — | 0.94 (*P* < 2.22 × 10^−16^) | 0.95 (*P* < 2.22 × 10^−16^) | 0.16 (*P* = 3.94 × 10^−10^) | 0.85 (*P* < 2.22 × 10^−16^) | 0.54 (*P* < 2.22 × 10^−16^) |
|  | **In- vs. Post-puberty** | 0.99 (*P* < 2.22 × 10^−16^) | — | 0.93 (*P* < 2.22 × 10^−16^) | 0.17 (*P* = 8.91 × 10^−12^) | 0.86 (*P* < 2.22 × 10^−16^) | 0.57 (*P* < 2.22 × 10^−16^) |
|  | **Pre- vs. Post-puberty** | 0.99 (*P* < 2.22 × 10^−16^) | 0.99 (*P* < 2.22 × 10^−16^) | — | 0.18 (*P* = 6.95 × 10^−14^) | 0.86 (*P* < 2.22 × 10^−16^) | 0.57 (*P* < 2.22 × 10^−16^) |
| **Densities of genes** | | 0.32 (*P* < 2.22 × 10^−16^) | 0.33 (*P* < 2.22 × 10^−16^) | 0.33 (*P* < 2.22 × 10^−16^) | — | 0.42 (*P* < 2.22 × 10^−16^) | 0.55 (*P* < 2.22 × 10^−16^) |
| **Densities of CGIs** | | 0.94 (*P* < 2.22 × 10^−16^) | 0.94 (*P* < 2.22 × 10^−16^) | 0.94 (*P* < 2.22 × 10^−16^) | 0.42 (*P* < 2.22 × 10^−16^) | — | 0.60 (*P* < 2.22 × 10^−16^) |
| **Densities of CpGs** | | 0.86 (*P* < 2.22 × 10^−16^) | 0.86 (*P* < 2.22 × 10^−16^) | 0.86 (*P* < 2.22 × 10^−16^) | 0.50 (*P* < 2.22 × 10^−16^) | 0.88 (*P* < 2.22 × 10^−16^) | — |

Correlation coefficients were calculated by Pearson’s correlation. The lower triangle represents the correlation coefficient of CpG methylomes, and the upper triangle represents the correlation coefficient of CpH methylomes.
